# Multi-Source Transfer Learning via Ensemble Approach for Initial Diagnosis of Alzheimer’s Disease

**DOI:** 10.1109/JTEHM.2020.2984601

**Published:** 2020-04-23

**Authors:** Yun Yang, Xinfa Li, Pei Wang, Yuelong Xia, Qiongwei Ye

**Affiliations:** 1 National Pilot School of SoftwareYunnan University 12635 Kunming650091China; 2 School of Information Science and EngineeringYunnan University 12635 Kunming650091China; 3 School of BusinessYunnan University of Finance and Economics 66569 Kunming650221China

**Keywords:** Alzheimer’s disease, multi-source transfer learning, ensemble learning, auxiliary diagnosis system

## Abstract

Alzheimer’s disease (AD) is one of the most common progressive neurodegenerative diseases, and the number of AD patients has increased year after year with the global aging trend. The onset of AD has a long preclinical stage. If doctors can make an initial diagnosis in the mild cognitive impairment (MCI) stage, it is possible to identify and screen those at a high-risk of developing full-blown AD, and thus the number of new AD patients can be reduced. However, there are problems with the medical datasets including AD data, such as insufficient number of samples and different data distributions. Transfer learning, which can effectively solve the problem of distribution discrepancy between training and test data and an insufficient number of target samples, has attracted increasing attention over recent years. In this paper, we propose a multi-source ensemble transfer learning (METL) approach by introducing ensemble learning and our tri-transfer model that uses Tri-Training, which ensures the transferability of source data by the tri-transfer model and high performance through ensemble learning. The experimental results on the benchmark and AD datasets demonstrate that our proposed approach has effective transferability, robustness, and feasibility, and is superior to existing algorithms. Based on METL, we propose an auxiliary diagnosis system for the initial diagnosis of AD, which helps doctors identify patients in the MCI stage as quickly as possible and with high accuracy so that measures can be taken to prevent or delay the occurrence of AD.

## Graphical abstract

This paper presents an auxiliary diagnosis system, based on multi-source ensemble transfer learning (METL), for the initial diagnosis of Alzheimer's disease, which helps doctors identify patients in the mild cognitive impairment stage as quickly as possible and with high accuracy so that measures can be taken to prevent or delay the occurrence of Alzheimer's disease.

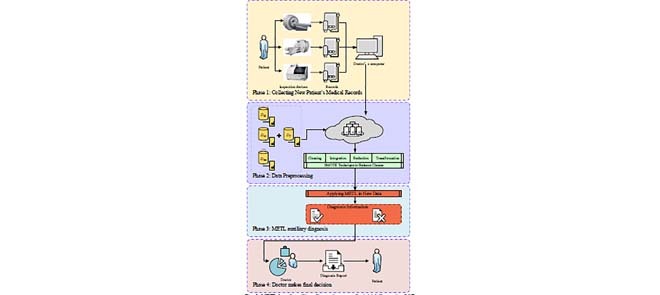

## I. Introduction

Machine learning has shown great success in variety of application fields, including computer vision, object recognition, and natural language processing [1], [2]. Some scholars have applied machine learning in the medical field, which led to the emergence of machine learning-driven intelligent auxiliary diagnostic systems [3], [4].

Alzheimer’s disease (AD) is one of the most common progressive neurodegenerative diseases, and with the global aging trend, the number of patients with AD has increased year after year. It is estimated that by 2050, AD patients will increase by three times [5]. Medical research shows that in the early stage of AD, patients will present with mild cognitive impairment (MCI) [6], which lies between the normal state and the diseased state and begin to appear younger patients. Many studies are based on the hope that potential AD patients can be detected during the MCI stage, and then effective measures can be taken to prevent the disease from worsening. If early prevention and treatment are available, the number of new patients will be reduced. If the MCI stage can be studied in depth, it is hoped that the high-risk population of AD will be discovered and screened, thus providing an optimal treatment time window for preventing or delaying the occurrence of AD. The Alzheimer’s Disease Neuroimaging Initiative (ADNI) [7] provides researchers committed to determining the progression of AD with research data. ADNI research resources and data include MRI images, PET images, genetic data, and clinical data from the North American ADNI Study; the collected samples include patients with Alzheimer’s disease, subjects with mild cognitive impairment, and elderly controls.

Traditional machine learning still suffers from two defects: 1) high labor intensity for labeled data, especially for insufficient AD samples and 2) different data distributions that produced different regions and ages, multi-source medical datasets such as MRI images, PET images, genetic data, clinical data. Due to the above problems, it is difficult to obtain accurate classifiers directly by using traditional machine learning. Transfer learning was proposed to address these issues by imitating the learning of human beings. The core idea of transfer learning is to transfer knowledge from a well-trained source domain to a target domain where training data is insufficient. Due to its advantages, transfer learning has been widely used in various cross-domain fields and has been attracting increasing attention in recent years [8], [9].

The key issue in transfer learning is inappropriate domain adaption that results from different data distributions across domains [10]. Moreover, some transfer will not improve performance or may even reduce the performance of the target classifier; this is called negative transfer [11]. Many approaches aim to address this issue, such as TrAdaBoost and Co-Clustering approaches, and a comprehensive review on transfer learning is given in [10]. However, most existing approaches, such as TrAdaBoost [12] and Co-Clustering [12], only utilize a single source domain. In actual medical problems, the target domain often involves knowledge from multiple source domains. Therefore, transfer learning involving multiple source domains, referred as multi-source transfer learning (MSTL), is proposed to effectively utilize the knowledge from different domains [13], [14].

Multiple source domains not only bring benefits but also a new challenge, i.e., how to identify and select the useful knowledge from multiple source domains. The knowledge from multiple source domains usually have different distributions, and thus not all knowledge can be reused to improve performance. Thus, inappropriate selection and deployment of source domains will exacerbate negative transfer [13]. Several approaches were proposed to address this issue [13], [14]. Although these approaches have been developed to alleviate the limitation of negative transfer, it could reduce performance because most of them are unattachable to explore the distribution similarity between source and target domains, and to handle the imbalanced data.

In this paper, we propose a multi-source ensemble transfer learning (METL) approach. METL consists of two phases: (1) single-source tri-transfer learning, which improves the transferability of the classifier trained by a single source domain, and (2) MI-based multi-source ensemble learning, which ensembles multiple classifiers into a robust final classifier. To validate METL, we conduct four sets of experiments via a variety of multi-source transfer tasks. The experimental results show that METL outperforms existing algorithms in medical fields and has practical capability in AD initial diagnosis. To further prevent or delay the occurrence of AD, we propose an METL-based auxiliary diagnosis system, which helps doctors to identify patients in MCI stage as quickly and accurately as possible.

The rest of this paper is organized as follows. Section II discusses the related work. Section III presents the details of our approach. Section IV reports on and analyzes the experimental results on benchmark and AD datasets. Section V discusses the results, the limitations of our approach, and future work. Finally, Section VI concludes this paper.

## II. Related Work

In recent years, many improved transfer learning algorithms have been proposed by combining with other methods. In this part, we discuss algorithms related to our work.

### A. Transfer Learning Based on Ensemble Learning

Ensemble learning occurs when tasks are learned by combining the strengths of a collection of simpler base models [15]–[17]. In general, ensembled learners outperform the single algorithm in three aspects: (1) Accuracy: An ensembled solution has better average performance. (2) Novelty: An ensembled solution is unattainable by any single algorithm. (3) Robustness: An ensembled solution has lower sensitivity to noise, outliers, or sampling variations. Dai *et al.* [12] proposed a classic correlation-based TrAdaBoost algorithm, which reasonably adjusted the weights of examples. Liu [18] presented a transfer learning algorithm that dynamically reassembled the main training dataset, and quickly eliminated redundant data. Xiao *et al.* [19] proposed a dynamic transfer ensemble model based on clustering and selection. Meanwhile, Mei [20] proposed a transfer learning framework for large-scale membrane protein identification based on the SVM ensemble.

### B. Multi-Source Transfer Learning

Yao and Doretto [13] proposed Multi-Source-TrAdaBoost (MTrA), which extends TrAdaBoost to utilize multiple sources. However, MTrA selects only one source domain that is closest related to the target domain at each iteration. Qian *et al.* [14] proposed an algorithm based on multi-sources dynamic TrAdaBoost (MSDTrA), which ensembles all knowledge, but it does not consider unbalanced classes. Ge *et al.* [21] proposed the Supervised Local Weight (SLW) method, which effectively transfers knowledge even if there are unrelated source domains and unbalanced classes; however, it is not applicable to the classification of high-dimension data. Eaton and Desjardins [22] presented a novel set-based boosting technique that boosts each source task and assigns higher weights to source tasks with positive transferability.

### C. Transfer Learning for Ad Auxiliary Diagnosis

Cheng *et al.* [23] presented a novel domain transfer learning approach for MCI conversion prediction, which contains three transfer components and uses data from both the target domain (i.e., MCI) and source domains (i.e., AD and normal control). Since 2D convolutional neural networks (CNN) will not be able to consider the relationship between 2D image slices in the MRI volume and make decisions on them independently. Ebrahimi-Ghahnavieh *et al.* [24] proposed to utilize recurrent neural network after the CNN and transfer learning to understand the relationship. Li *et al.* [25] presented an effective knowledge transfer method is proposed to reduce the differences between different data sets and improve the classification accuracy of data sets with insufficient training samples, tested on a small dataset from a local hospital and a large shared dataset.

## III. Multi-Source Ensemble Transfer Learning

**FIGURE 1.**
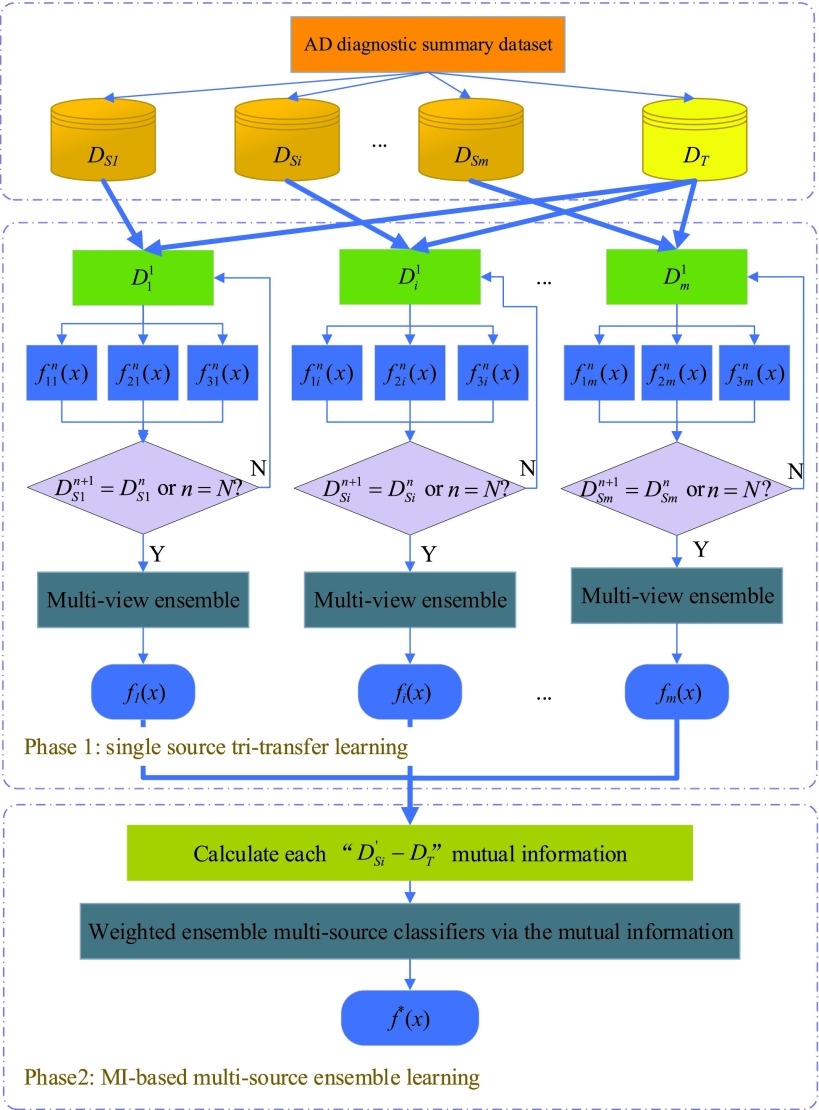
Multi-source ensemble transfer learning approach.

In this section, we describe the details of METL. The framework of METL is shown in Fig. 1. METL consists of two phases: single-source tri-transfer learning and mutual information-based (MI-based) multi-source ensemble learning. Single-source tri-transfer learning improves the transferability of the classifier trained by a single source domain, while MI-based multi-source ensemble learning combines multiple classifiers into a final robust classifier.

According to the definition of transfer learning, data in the source domain $D_{S} $ has the same feature space $X$ as data in the target domain $D_{T} $ but has a different data distribution. $D_{S} =\{(x_{1}^{S},y_{1}^{S} \},\cdots,(x_{m}^{S},y_{m}^{S})\}$, where $x_{i}^{S} \in X_{S} $ is an instance, and $y_{i}^{S} \in Y_{S} $ is the corresponding label. $D_{T} =\{(x_{1}^{T},y_{1}^{T}),\cdots,(x_{n}^{T},y_{n}^{T})\}$, where $x_{i}^{T} \in X_{T} $ is an instance, and $y_{i}^{T} \in Y_{T} $ is the corresponding class label. In our approach, substantial labeled examples are available in source domains, and a few labeled examples are useful in the target domain.

**Phase 1 (single source tri-transfer learning):**

At this phase, one source domain (i.e., one of $D_{S1},D_{S2,} D_{Si},\cdots,D_{Sm}$) and target domain $D_{T} $ are first combined to generate a new training dataset $D_{1},D_{2},D_{i},\cdots,D_{m} $. Then, three heterogeneous classifiers are iteratively trained on the new training dataset until a metric is satisfied. Here, we propose a novel source data sample method to effectively sample high-confidence data from source domains. As soon as the iterations stop, the three classifiers are ensembled to generate a robust classifier for one source domain, e.g., $f_{1} (x),f_{2} (x),f_{3} (x)$. The main object of this phase is to enhance the transferability from one source domain to the target domain. (Details are in Section III.A)

**Phase 2 (MI-Based multi-source ensemble learning):**

After phase 1, many classifiers are obtained, each corresponding to one source domain. We propose a novel approach to weigh these classifiers based on the correlation between the source domain and the target domain. By means of our proposed weight assignment, each source classifier is given an optimal weight. Finally, all classifiers are ensembled to generate the final classifier $f^{\ast }(x)$ for the target domain. (Details in Section III.B)

### A. Single-Source Tri-Transfer Learning

Tri-Training [26] is a semi-supervised learning algorithm that uses three different classifiers to exploit unlabeled data for enhancing learning performance. Inspired by Tri-Training, we derive three heterogeneous classifiers $f_{1i},f_{2i},f_{3i} $ from different “views,” i.e., using different features. In phase 1, the core concept is to check the consistency between these classifiers. We assume that if they have the same predication for one instance $x_{j} $, the transferability of $x_{j} $ is considered to be high and should be included to improve the prediction performance for the target domain. Different from the Tri-Training bootstrap sampling mechanism, where it is meaningless to divide a source domain into multiple source domains with the same data distribution, single-source tri-transfer learning employs a new source data sampling method for the multi-view ensemble. Here, we can improve the transferability of a single source domain to the target domain, thus avoiding negative transfer.

Softmax [27], Support Vector Machine (SVM) [28], and Deep Neural Network (DNN) [29] are chosen as our three heterogeneous base classifiers. The Softmax classifier is a linear classifier, the input is an example feature, and the output is the probability that the example belongs to each category, which is flexible, efficient, and time-saving. SVM is an algorithm that uses nonlinear mapping to transform low-dimensional training data into higher dimensions, which builds an optimal hyperplane in feature space based on structural risk minimization theory. Therefore, it is robust, accurate, and less prone to overfitting. Finally, DNN mimics the learning mechanism of the brain, automatically combining simple features into more complex features, and uses these combined features to solve problems. Thus, the DNN has strong generalization ability.

Therefore, we can identify all useful data sample with high confidence by checking the predictive consistency of three heterogeneous classifiers. However, checking the consistency between three classifiers only once may not sample a good source data for transfer learning. Furthermore, we use an iterative approach to refine the data samples of the source domain.

The pseudo-code of phase 1 is given in Algorithm METL. As shown in Algorithm METL, we initially combine the target training dataset $D_{T} $ with data in the $i$-th source domain $D_{Si} $ to form a new training dataset $D_{i}^{1} $. Three classifiers are given to train $D_{i}^{1} $ from different views. We sample all examples with consistent results from three classifiers into $D_{Si}^{n+1} $. Then, $D_{Si}^{n+1} $ and $D_{T} $ form a new training set. We update the three classifiers and repeat the above-mentioned steps. The algorithm terminates when the training dataset is no longer changed and finally outputs the latest classifiers.

Once the final classifiers are derived, we use a multi-view ensemble method to train a more robust classifier for one source domain. The strong classifier of the *i-* th source domain is denoted as $f_{i} (x)$ and can be calculated as follows:

\begin{equation*} f_{i} (x)=\sum \nolimits _{k=1}^{3} {\frac {1}{3}f_{ki}^{n} (x)}\tag{1}\end{equation*}

### B. MI-Based Multi-Source Ensemble Learning

After the first step, we have obtained one classifier for each source domain. Due to use one single classifier is unlikely to provide a robust classifier for the target domain, but ensemble learning can improve this by combing several classifiers. In ensemble learning, we need to weigh ensemble classifiers according to their correlation such that the final classifier achieves the best performance. Likewise, we utilize ensemble learning to combine all classifiers from the source domains to produce a more robust and predictive classifier for the target domain.

Inspired by the distribution weighted combination rule [30], the ideal target classifier can be treated as a mixture of multiple source classifiers weighted by normalized source distributions. In other words, the multi-source transfer learning problem is viewed as finding the “mean” predicted labels of all possible predicted labels that are generated by the corresponding source classifiers.

The pseudo-code of phase 2 is given in Algorithm METL. In phase 2, we select mutual information to assign different classifier weights. Mutual information from information theory [31] is widely used to describe the mutual dependence between two random variables. In METL, different source domains and target domains may have diverse data distributions. The source domains with a similar data distribution as the target domain should contribute more in our ensemble learning in terms of improving performance.

As mentioned above, $p(x,y)$ denotes the joint distribution of two random variables $(X,Y)$, while $p(x)$ and $p(y)$ denote the edge distribution of $X$ and $Y$, respectively. The mutual information of $X$ and $Y$ is expressed as $I(X;Y)$, which is the relative entropy of $p(x,y)$ and the distribution product $p(x)p(y)$, as shown in Eq. (2).

\begin{equation*} I(X;Y)=\sum \nolimits _{x\in X} {\sum \nolimits _{y\in Y} {p(x,y)}} \log \frac {p(x,y)}{p(x)p(y)}\tag{2}\end{equation*}

The mutual information value between the source sample $x_{m}^{S_{i}^{\prime }} $ in the *i-*th source domain after iterations $D_{Si}^{\prime } $, and the target sample $x_{n}^{T} $ is obtained from Eq. (3).

\begin{equation*} I(x_{m}^{S_{i}^{\prime }};x_{n}^{T})=\sum \nolimits _{x\in x_{m}^{S_{i}^{\prime }}} {\sum \nolimits _{y\in x_{n}^{T}} {p(x,y)\log \frac {p(x,y)}{p(x)p(y)}}}\tag{3}\end{equation*}

For $D_{Si}^{\prime } $ and $D_{T} $, the mutual information value between the two data distributions is calculated by Eq. (4), which actually computes the mean of all relevant source and target samples:

\begin{equation*} I(D_{Si}^{\prime }; D_{T})=\overline {I(x_{m}^{S_{i}^{\prime }};~x_{n}^{T})}\tag{4}\end{equation*}

We use mutual information $I(D_{Si}^{\prime },D_{T})$ to indicate the weight of one source domain $D_{Si} $ and target domain $D_{T} $. Hence, for each source domain $D_{Si} $, we have weight $w_{i} =I(D_{Si};D_{T})$. We normalize weight $w_{i}^{\ast } $ as follows:

\begin{equation*} w_{i}^{\ast } =\frac {w_{i}}{\sum \nolimits _{k=1}^{m} {w_{k}}}\tag{5}\end{equation*}

where $w_{i}^{\ast } \in [{0,1}]$ and $\sum \nolimits _{i=1}^{m} {w_{i}^{\ast }} =1$. The target classifier is treated as a linear combination of the multiple classifiers with a weight $w_{i}^{\ast } $, and weights for all source classifiers collectively form a weight vector ${\mathbf { w}}^{\ast }=\left \{{{w_{i}^{\ast }} }\right \}_{i=1}^{m} $. Finally, we utilize the value of weighted ensemble classifiers from multiple source domains and obtain an ensemble transfer learning effect with high performance and robustness. According to the above description, the function of the final classifier $f^{\ast }(x)$ is described as follows:

\begin{equation*} f^{\ast }(x)=\sum \nolimits _{i=1}^{m} {w_{i}^{\ast } f_{i} (x)}\tag{6}\end{equation*}

### C. AD Initial Diagnosis With METL

**FIGURE 2.**
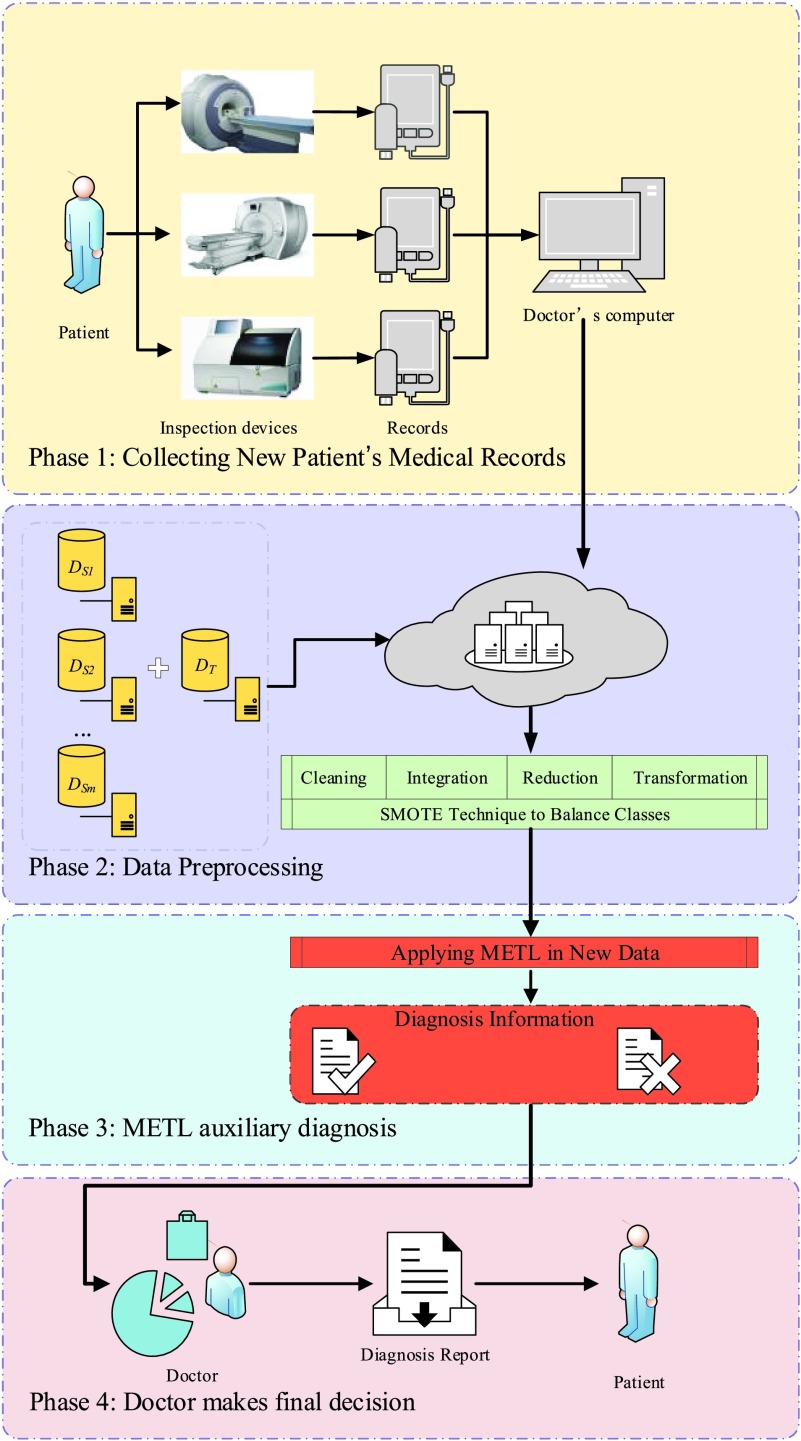
METL-based auxiliary diagnosis system for initial diagnosis of AD.

We combine the proposed approach with the traditional medical diagnosis process to achieve practical application value. The ultimate goal is to help solve medical problems and facilitate early diagnosis of AD. The METL-based auxiliary diagnosis system is shown in Fig. 2; the system simulates the traditional diagnosis process. It has four phases: collecting the new patient’s medical records, data preprocessing, METL auxiliary diagnosis, and final diagnosis by the doctor.

The first phase is to use medical devices to examine the new patient, collecting information such as MRI images, PET images, and clinical data. Then, we generate an inspection report, present this report to the patient, and upload the data to doctors’ computers and servers. The second phase is the preprocessing of the new patient data and source and target domain datasets that from ADNI, including cleaning, integration, reduction, transformation, and class balancing. The third phase aims to generate an METL classification model and use the model to generate an auxiliary diagnosis; the results are displayed as either healthy or sick, the latter meaning the patient is in the MCI stage. In the fourth phase, the doctor refers to the auxiliary diagnosis result, makes the diagnosis, and informs the patient.

Different from the traditional diagnosis process, the proposed METL-based auxiliary diagnosis system not only reduces human error, but also improves accuracy, enabling doctors to make accurate judgments as soon as possible. If patients are found to be in the MCI stage, then the occurrence of AD can be prevented or delayed, thereby reducing the number of AD patients [32].

### D. Theory Analysis

#### 1) Phase 1 (Single Source Tri-Transfer Learning)

Let $p^{S_{k}}(x),p^{S_{k}}(y\vert x),p^{S_{k}}(x,y)$ denote the marginal, conditional, and joint distribution of the source domains, respectively, and $p^{T}(x),p^{T}(y\vert x),p^{T}(x,y)$ for those of the target domain. It is obvious that if the prediction of classifier $f_{1},f_{2},f_{3} $ for the source sample $x_{i}^{S_{K}} $ is the same, then this source sample is deemed to have a highly similar distribution with the target domain and is marked with a high confidence value, and vice versa. Here, we use $\beta _{i} $ to represent the transferability of one source sample $x_{i}^{S_{K}}$, which is defined in Eq. (7).

\begin{equation*} \beta _{i} =\exp [\alpha _{i} p^{T}(x_{i}^{S_{k}})]\tag{7}\end{equation*}

Here, $p^{T}(x_{i}^{S_{k}})$ denotes the probability of sample $x_{i}^{S_{K}} $ generated under the target domain distribution. $\alpha _{i} $ is an indicator of whether three classifiers have the same predication as $f_{1} (x_{i}^{S_{k}})=f_{2} (x_{i}^{S_{k}})=f_{3} (x_{i}^{S_{k}})$. If so, $\alpha _{i} =1$; otherwise, $\alpha _{i} =-1$.

The large distribution difference between the source and target domains is an important factor of negative transfer. To eliminate the distribution difference between source and target domains, we weigh the source sample with its transferability. $\hat {p}^{S_{k}}(x,y)=\beta p^{S_{k}}(x,y)$ is defined as the estimated joint distribution of the source domain. Based on the Kullback–Leibler (KL) divergence [33], we define the following objective function for minimizing the distribution difference:

\begin{align*} KL[p^{T}(x,y)\vert \vert \hat {p}^{S_{k}}(x,y)]=&\int \!\!\!\int _{D} {p^{T}(x,y)} \log \frac {p^{T}(x,y)}{\hat {p}^{S_{k} }(x,y)}\textrm {d}x\textrm {d}y \\=&\int \!\!\!\int _{D} {p^{T}(x,y)\log \frac {p^{T}(x,y)}{p^{S_{k} }(x)}\textrm {d}x\textrm {d}y} \\&-\!\Biggl ({\! {\int \!\!\!\int _{D} {p^{T}(x,y)\log p^{S_{k}}\left ({{y\vert x} }\right)} \textrm {d}x\textrm {d}y}} \\&+\,{{\int \!\!\!\int _{D} {p^{T}(x,y)\log \beta } \textrm {d}x\textrm {d} y} }\Biggr)\tag{8}\end{align*}

The objective function contains two terms, and the first term is fixed when the dataset is known. Hence, to minimize Eq. (8), we just need to maximize the second part (within the parentheses). The second term can be maximized by training a better classifier. Consequently, optimizing Eq. (8) is equivalent to maximizing the third term, which becomes

\begin{align*}&\hspace {-1.2pc}\max \sum \nolimits _{i=1}^{n_{S_{k}}} {\log \beta _{i}^{t}} \\=&\sum \nolimits _{i\in D_{S_{k}}^{+}} {\alpha _{i}} p^{T}(x_{i}^{S_{k}}) +\sum \nolimits _{i\in D_{S_{k}}^{-}} {\alpha _{i} p^{T}} (x_{i}^{S_{k}}) \\=&\sum \nolimits _{i\in D_{S_{k}}^{+}} {p^{T}} (x_{i}^{S_{k}}) -\sum \nolimits _{i\in D_{S_{k}}^{-}} {p^{T}(x_{i}^{S_{k}})} \\=&s_{i}^{+} -s_{i}^{-}\tag{9}\end{align*}

where $D_{S_{k}}^{+} \cup D_{S_{k}}^{-} =D_{s_{k}} $, and $D_{S_{k} }^{+} \cap D_{S_{k}}^{-} =\emptyset $. $D_{S_{k}}^{+} $ denotes the set of examples on which $f_{1} (x_{i}^{S_{k}})=f_{2} (x_{i}^{S_{k}})=f_{3} (x_{i}^{S_{k}})$, and $D_{s_{k}}^{-} $ denotes the rest of the source examples. Moreover, $s_{i}^{+} =\sum \nolimits _{i\in D_{S_{k}}^{+}} {p^{T}} (x_{i}^{S_{k}})$, and $s_{i}^{-} =\sum \nolimits _{i\in D_{S_{k}}^{-} } {p^{T}} (x_{i}^{S_{k}})$, which means that sample $x_{i}^{S_{k}} \in s^{+}$ is helpful for learning the target task. In contrast, when $x_{i}^{S_{k}} \in s^{-}$, it plays a negative role. Note that $s_{i}^{+},s_{i}^{-} \ge 0$, and we can maximize the function as show in (9) by selecting better transferability of source samples $x_{i}^{S_{k}} \in s^{+}$.

#### 2) Phase 2 (MI-Based Multi-Source Ensemble Learning)

In phase 2, we denote $f^{\ast }(X^{T})$ and $\{f_{i} (X^{T})\}_{i=1}^{m} $ as the target labels predicted by the ideal target classifier and the source classifier, respectively. As mentioned above, the ideal target classifier can be derived by minimizing the loss function:

\begin{equation*} L=\sum \nolimits _{i=1}^{m} {w_{i}} d(f^{\ast }(X^{T}),f_{i} (X^{T}))\tag{10}\end{equation*}

where $w_{i} $ refers to the weight of the source classifier $f_{i} (x)$, and $d$ is a distance metric approach. A classification function $f(\cdot)$ can be written as $p(y\vert x)$ from a probabilistic viewpoint. For each source classifier, the predicted labels $f_{i} (X^{T})$ are mathematically represented as probability distributions: $p_{i} (x_{n})=\sum \nolimits _{y} {p_{i} (y)p_{i} (x_{n} \left |{ y }\right.)}$, where $p_{i} (y)$ is the prior probability of labels, and $p_{i} (x_{n} \vert y)$ is the post-probability of instance $x_{n}$. Using the KL distance, the loss function $L$ can be further derived as follows:

\begin{align*} L=&\sum \nolimits _{i=1}^{m} {w_{m}} D_{KL} (f_{i} (X^{T}),f^{\ast }(X^{T})) \\=&\sum \nolimits _{i=1}^{m} {w_{i} \sum \nolimits _{j=1}^{n} {p_{i} (x_{j})\log \frac {p_{i} (x_{j})}{p^{\ast }(x_{j})}}} \\=&\sum \nolimits _{i=1}^{m} {w_{i}} \sum \nolimits _{j=1}^{n} {\sum \nolimits _{y} {p_{i} (y)p_{i} (x_{j} \vert y)\log \frac {p_{i} (x_{j})}{p^{\ast }(x_{j})}}} \\=&\sum \nolimits _{i=1}^{m} {w_{i}} \sum \nolimits _{y} {p_{i} (y)} \left [\sum \nolimits _{j=1}^{n} {p_{i} (x_{j} \vert y)\log \frac {p_{i} (x_{j})}{p^{\ast }(x_{j})}} { }\right] \\=&\sum \nolimits _{i=1}^{m} {w_{i}} \sum \nolimits _{y} {p_{i} (y)} \Biggl [{-H}(p_{i} (x_{j} \vert y),p_{i} (x_{j}) ) \\& { {+\,H(p_{i} (x_{j} \vert y),p^{\ast }(x_{j}))} }\Biggr]\tag{11}\end{align*}

where $H(X)=-\sum \nolimits _{n} {p(x_{n})\log p(x_{n})} $ is the entropy, which is an uncertain property. The loss function $L$ can be further divided into two parts $L_{1} $ and $L_{2} $:

\begin{align*} L_{1}=&\sum \nolimits _{i=1}^{m} {w_{i}} \sum \nolimits _{y} {p_{i} (y)} \left [{ {-H(p_{i} (x_{j} \vert y),p_{i} (x_{j}))} }\right] \\ L_{2}=&\sum \nolimits _{i=1}^{m} {w_{i}} \sum \nolimits _{y} {p_{i} (y)} \left [{ {H(p_{i} (x_{j} \vert y),p^{\ast }(x_{j}))} }\right],\tag{12}\end{align*}

The performance of the ensemble learning approach depends on the predicted results of both the source classifier and the ensemble classifier. With the decrease of $L_{1} $, the source classifier can achieve better performance. The loss function $L_{1} $ defined in Eq. (12) refers to the confidence of the classification results. Since the information entropy is the confusion property for a system, better classification results have smaller dissimilarity. For $L_{2} $ show in Eq. (12), in order to guarantee the performance of the ensembled classifier, the member of ensembled classifier should have a higher accuracy and dissimilarity for the classification task.

## IV. Experimental Evaluation

To validate METL, we conduct extensive evaluations and experiments via a variety of multi-source transfer tasks. We first use a standard benchmark dataset to evaluate the following: (i) the efficacy of individual single-source tri-transfer learning and multi-source ensemble learning, (ii) the transferability of our approach, and (iii) the classification performance of our approach in comparison with other algorithms. Then, we use the AD dataset from ADNI to verify the feasibility of our proposed approach. Through these experiments, we comprehensively evaluate the performance of METL and the practical application capabilities in AD diagnosis.

### A. Benchmark Datasets

We first conducted experiments on 12 representative medical datasets from the UCI repository [34]. These 12 datasets, widely used for comparison between different algorithms, represent diverse domains and data features and have been preprocessed.

To form multiple sources for our problem, we divide each dataset from UCI into four sets, i.e., one target domain and three source domains. We select a multi-valued attribute and use K-means [35] on one attribute to cluster data into four sets, each set corresponding to one domain. The resultant four domains have different data distributions. TABLE 1 presents the attribute that is used to split each dataset and the details of each domain after splitting.

**TABLE 1:** Splitting Attributes and Partitions of the 12 UCI Datasets

Dataset	Attribute	Number of instances in different domains			
		$D_{T}$	$D_{S1}$	$D_{S2}$	$D_{S3}$
breast-cancer	Clump Thickness	363	246	183	326
diabetes	Preg	350	160	200	58
diabetic retinopathy	Numeric	481	253	89	328
heart-c	Cp	142	49	83	22
heart-statlog	Age	85	75	47	63
hepatitis	Age	54	42	22	36
ILPD	Age	263	239	220	96
mammographic_masses	Age	334	106	236	154
sani	Age	120	82	144	82
sick	T4U	1174	685	664	120
thoracic surgery	PRE4	151	120	76	123
wdbc	Radius 03	197	113	179	80

#### 1) Evaluation of the Two Phases

In order to demonstrate the effectiveness of the two phases of METL, we design two baseline approaches for comparison against METL. The first approach (prototype1) uses TrAdaBoost to replace the proposed tri-transfer model, and the SVM is selected as the basic classifier. The second approach (prototype2) replaces the MI-Based ensemble method with an equal-weighted ensemble method.

Algorithm 1 **METL**
Input: Source domain data $D_{Si} $ and target domain dataset $D_{T} $ with labels $y_{i} \in Y$.
Phase 1: $D_{i}^{1} \leftarrow D_{Si} \cup D_{T} $
**for** $n = 1$ to $N$ **do**
Using three heterogeneous classifiers, $f_{1i}^{n} (x)$, $f_{2i}^{n} (x)$ and $f_{3i}^{n} (x)$, to train on data $D_{i}^{n} $.
initialize $D_{Si}^{n+1} =\emptyset $.
$D_{Si}^{n+1} \leftarrow $ training on all instances in $D_{Si}^{n} $.
**if** $D_{Si}^{n+1} =D_{Si}^{n} $ **then**
break.
**end if**
$D_{i}^{n+1} \leftarrow D_{S_{i}}^{n+1} \cup D_{T} $
**end for**
$f_{i} (x)\leftarrow $ multi-view-ensemble three classifiers as Eq. (1).
Phase 2: **for** $i = 1$ to $m$ **do**
$f_{i} \leftarrow $ phase $1(D_{Si},D_{T})$
$w_{i} \leftarrow \sum \nolimits _{x\in x_{m}^{S_{i}^{\prime }}} {\sum \nolimits _{y\in x_{n}^{T}} {p(x,y)\log \frac {p(x,y)}{p(x)p(y)}}} $ as Eq. (3) and (4).
**end for**
$W^{\ast }\leftarrow \left\{{\forall i\in m\vert \frac {w_{i}}{\sum \nolimits _{k=1}^{m} {w_{k}}}}\right\}$ as Eq. (5).
$f^{\ast }\leftarrow \sum \nolimits _{i=1}^{m} {w_{i}^{\ast } f_{i} (x)} $ as Eq. (6)
Output: $f^{\ast }(x)$

Experiments using the three approaches are conducted on the 12 medical datasets. For comparison purpose, we chose 70% of the labeled examples in the target domain as the test dataset, and 3%, 10%, and 30% of the remainder as the training data. The number of source domains is 3. The experiments are repeated 10 times, and we average the results to obtain an accurate error estimate.

The experimental results are summarized in TABLE 2. We can see that METL outperforms the two baseline approaches in the majority of cases. The results prove that tri-transfer learning is generally better than TrAdaBoost, and the MI-based ensemble method generally surpasses the equal-weighted ensemble method. As the percent of labeled data in the target domain increases, the accuracy of three approaches is improved. Furthermore, as indicated by the accuracy on mammographic_masses and sani datasets, prototype1 outperforms METL. This is because in the case of 3% and 10% labeled training data in the target domain, only a few source samples can be obtained; tri-transfer learning may discard some of the samples are still useful even if they are not strongly correlated with the target domain. Therefore, the sampled source data may cause underfitting, and the value of mutual information is not able to correctly measure the similarity of data distributions between the source domain and target domain. When the percent of labeled data in the target domain reaches 30%, prototype1 and prototype2 are worse than METL, which means that METL classification performance is improved when the amount of training data is sufficient.

**TABLE 2:** Classification Accuracy of Different Prototypes on the 12 UCI Datasets

Dataset	3%			10%			30%		
	prototype1	prototype2	METL	prototype1	prototype2	METL	prototype1	prototype2	METL
breast-cancer	97.52	97.06	98.16	98.78	98.07	98.89	99.08	98.8	99.17
diabetes	75.84	81.95	81.9	79.42	83.09	83.8	81.71	83.17	85.71
diabetic retinopathy	62.56	62.5	63.05	63.47	63.36	63.75	67.76	67.15	67.78
heart-c	81.93	83.72	86.82	80.71	84.49	88.37	86.53	97.98	90.69
heart-statlog	69.7	70.83	70.21	74.3	74.75	75	77.98	82.02	84.61
hepatitis	80.88	79.73	82.35	82.35	81.04	84.31	83.72	82.35	88.23
ILPD	73.29	74.93	74.68	77.85	78.1	78.35	77.97	78.23	78.48
mammographic_masses	63.4	63	63.2	64.5	63.9	64.11	81	80.5	81
sani	82.78	80.28	81.94	95.55	94.17	94.72	96.38	95.83	97.22
sick	96.03	96.03	96.03	95.65	96.31	96.6	97.28	97.45	97.73
thoracic surgery	82.94	83.47	84.13	90.93	91.03	91.73	91.33	91.87	92
wdbc	91.52	91.69	92.54	95.93	96.61	96.76	98.88	99.15	99.32
Average	78.39	79	79.83	81.26	81.73	82.79	84.94	86.35	87.06

#### 2) Evaluation of Multiple Sources

To verify the transferability of our approach, i.e., that it not only makes full use of data in the multiple source domains when there is little labeled data in the target domain but also avoids negative transfer, we conduct evaluations with multiple sources. We choose 3%, 10%, and 30% of labeled data in the target domain, with 0, 1, 2, and 3 source domains. We employ METL with different ratios of labeled data and different numbers of source domains, and then repeat the experiments 10 times and average the results.

As shown in TABLE 3, the average classification accuracy of the case with 3% labeled data and zero source domains is the worst while the case with 30% labeled data and three source domains is the best. In general, with the increasement in the ratio of target labeled data, the accuracy of METL increases. This means that an increase in the amount labeled data in the target domain more fully describes the data distribution, and hence the three heterogeneous classifiers have better generalization ability to ensure that the examples sampled from source domains have transferability. Furthermore, the accuracy raises with the increase in the number of source domains, indicating that METL can use samples from multiple source domains to assist learning the target task.

**TABLE 3:** Classification Accuracy on the 12 UCI Datasets With Different Number of Source Domains

Dataset	0			1			2			3		
	3%	10%	30%	3%	10%	30%	3%	10%	30%	3%	10%	30%
breast-cancer	81.44	91.43	95.41	94.95	98.17	98.53	95.57	98.69	99.08	98.16	98.89	99.17
diabetes	75.84	81.95	81.9	79.42	83.09	83.8	81.71	83.17	85.71	82.28	84.04	86.66
diabetic retinopathy	60.83	60.88	61.86	62.63	62.92	64.86	62.99	63.33	66.67	63.05	63.75	67.78
heart-c	81.39	82.17	82.17	83.72	84.49	85.27	86.04	86.82	87.98	86.82	88.37	90.69
heart-statlog	65.42	69.44	78.2	67.34	71.3	81.6	68.76	72.22	82.05	70.21	75	84.61
hepatitis	76.47	79.41	81.25	83.9	82.35	86.27	83.9	82.9	82.35	82.35	84.31	88.23
ILPD	66.67	71.14	73	69.16	72.91	78.35	73.67	77.97	78.41	74.68	78.35	78.48
mammographic_masses	37.5	63.1	69.67	63	63.8	75.5	63.09	63.9	77.4	63.2	64.11	81
sani	61.42	68.52	86.42	76.39	85.28	89.17	80.83	93.06	96.3	81.94	94.72	97.22
sick	94.8	96.03	96.77	95.93	96.22	96.69	96.03	96.37	97.26	96.03	96.6	97.73
thoracic surgery	81.44	91.43	95.41	94.95	98.17	98.53	95.57	98.69	99.08	98.16	98.89	99.17
wdbc	75.84	81.95	81.9	79.42	83.09	83.8	81.71	83.17	85.71	82.28	84.04	86.66
Average	70.18	76.41	80.87	77.64	80.05	84	79.26	81.84	85.32	79.87	82.81	87.16

The experimental results in TABLE 3 show that multi-source transfer learning outperforms single-source transfer learning. When the ratio of labeled data is 3%, the growth rate of the accuracy is the largest, which means that the less training data in the target domain, the more useful the transfer knowledge. However, when the ratios of labeled data are the same, the accuracy growth rate slowly decreases, which means that when there is enough source data, increasing the number of source domains will not significantly improve the performance.

#### 3) Comparison With Existing Approaches

To further demonstrate the performance of METL, we compare it with three transfer learning algorithms: MultiSource-TrAdaBoost (MTrA) [13], Multi-Source Dynamic TrAdaBoost (MSDTrA) [14], and Multi-Source Tri-Training Transfer Learning (MST^3^L) [36]. The main settings of algorithms are shown in TABLE 4.

**TABLE 4:** Main Sets of Multi-Source Transfer Learning Algorithms

Algorithm	Base Learner	Iteration	Ratio
METL	three classifiers	20	10%
MTrA	SVM	20	10%
MSDTrA	SVM	20	10%
MST^3^L	SVM	20	10%

To seek an accurate error estimate, each algorithm repeats cross-validation 10 times, and the mean is taken as the final result. As indicated by the average classification accuracy in TABLE 5, when the ratio of labeled data is 10%, METL is superior to MTrA, MSDTrA, and MST^3^L moreover, MTrA performs the worst. Three heterogeneous classifiers learn the same target task from different views, with strong generalization ability. Furthermore, METL reasonably estimates the correlation between each source and target domain by employing mutual information. MTrA and MSDTrA are both based on TrAdaBoost, but MSDTrA surpasses MTrA since MSDTrA joins dynamic factor improves the problem that the weight entropy caused by source weight convergence is transferred from the source sample to the target sample.

**TABLE 5:** Accuracy of Four Transfer Algorithms on the 12 UCI Datasets

Dataset	METL	MTrA	MSDTrA	MST^3^ L
breast-cancer	98.89	98.17	98.44	98.62
diabetes	84.04	83.8	83.94	84.28
diabetic retinopathy	63.75	63.26	64.24	63.13
heart-c	88.37	85.46	87.59	87.9
heart-statlog	75	74.16	74.16	75
hepatitis	84.31	83.42	83.66	84.45
ILPD	78.35	77.72	77.98	78.1
mammographic	64.11	63.4	65	63.4
sani	94.72	95	95.56	94.44
sick	96.6	93.93	94.35	96.69
thoracic surgery	98.89	98.17	98.44	98.62
wdbc	84.04	83.8	83.94	84.28
Average	82.81	81.83	82.49	82.6

### B. Alzheimer’s Disease Dataset

To further validate the feasibility of the proposed approach, we conduct extensive experiments on real-world AD medical dataset. More than 30 million people worldwide suffer from AD, and with the increase in life expectancy, patients are expected to triple by 2050. Medicine has shown that during MCI, timely detection and effective measures can prevent the disease from worsening. Therefore, the early diagnosis of AD is very important, and determining the patient’s stage in the disease has become the focus of current research.

ADNI provides researchers with research data as they work to determine the progression of AD. The data collection is divided into four phases: ADNI1, ADNI-GO, ADNI2, and ADNI3; ADNI3 is the latest stage. We used the AD diagnostic summary dataset obtained from ADNI, which includes the time phase, ID, multiple attributes of the inspection item, and diagnostic results labels. Attributes of the inspection item are DXCURREN, DXCONV, DXCONTYP, DXREV, DXNORM, DXMCI, DXMDES etc. We used data from the ADNI3 stage, including label 1 or 2, and then employed data preprocessing and SMOTE techniques [37] to balance the number of classes so that the training data in the AD dataset was easy to learn. The partitions of the AD dataset are shown in TABLE 6.

**TABLE 6:** Domain Partitions of the Dataset

Dataset	Instances			
	$D_{T}$	$D_{S1}$	$D_{S2}$	$D_{S3}$
AD	735	539	432	79

On the AD dataset, METL was compared with the three aforementioned algorithms. The main settings of the four algorithms are the same as those listed in TABLE 4. The ratios of labeled data in the target domain are selected as 3%, 10%, and 30%. The four algorithms are repeat cross-validation 10 times, and the experimental results are averaged.

**FIGURE 3.**
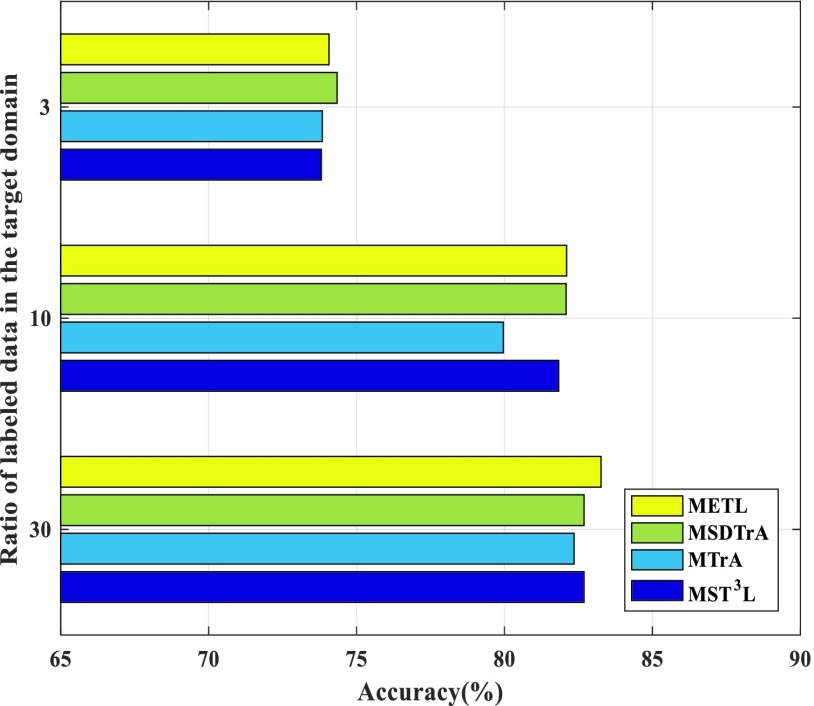
Classification accuracy of four algorithms on the AD dataset.

In Fig. 3, the x axis is the accuracy and the y axis is the ratio of the labeled data in the target domain. As indicated by the overall classification accuracy in Fig. 3, METL and MST^3^L are better than MSDTrA, but MTrA is worse than MSDTrA. When the ratio of labeled data in the target domain is 3%, METL and MST^3^L significantly outperform MTrA and MSDTrA, which demonstrates that METL has better transferability when there is very little training data in the target domain. MTrA and MSDTrA have similar accuracies when the ratio of labeled data in the target domain reaches 30%, which means that MTrA and MSDTrA have similar performance when the training data in the target domain is sufficient.

Moreover, Fig. 3 shows that METL has good feasibility in the initial diagnosis of AD and can help solve practical problems. As the ratio of labeled data in the target domain increase, the accuracies of the four algorithms will increase. However, the growth rate of accuracy from 3% to 10% of the ratio of labeled data in the target domain is higher than that from 10% to 30%. This means that the less labeled data there is in the target domain, the more useful the transfer learning.

## V. Discussion

As demonstrated in the reported experiments, our approach obtains a high-quality transfer performance. Based on the mathematical analysis and overall observation of the experimental results, we summarize the advantages of our approach as follows.

First, we proposed a single-source tri-transfer learning model that has been proved mathematically feasible in Sect III.D, and we tested it on a variety of datasets. The experimental results as shown in TABLE 2, TABLE 3, TABLE 5, and Fig. 3. The tri-transfer learning not only ensures that the sampled source data has better transferability compared to general transfer learning algorithms but also enhances robustness. Second, the MI-based ensemble method was initially proved feasible via a mathematical derivation and then demonstrated effectiveness of the method through experiments. Finally, experimental results show that our approach can assist initial diagnosis of AD.

Liu *et al.* [38] designed an ensemble transfer learning framework that uses a weighted resampling method on the source and target data. However, the framework is used for a single source domain, and their base learners are trained by the resampling method and TrAdaBoost. In contrast, METL learns three classifiers via the sampling scheme to ensure that the transferability of sampled source data. Therefore, our approach improves not only the interaction between multiple learners but also the reliability of source data.

Although the experimental results demonstrate that our approach achieves a certain level of superiority on a variety of datasets, there are some issues that could directly affect its practical application. Like all existing transfer algorithms, our approach may incur poor performance when the target examples are very few. Furthermore, while our approach chose Softmax, the SVM, and the DNN as the base learners, selecting appropriate classifiers for datasets with different data characteristics remains worthy of further research. Moreover, obtaining the shared feature space between the source and target domains will be a direction for our future work because of heterogeneous data in medical field.

The rapid aging of the population and the high incidence of chronic diseases, especially AD, are increasingly serious social problems worldwide. Through our approach, we can slow down and interfere with the clinical conversion of MCI or normal control to AD, thereby providing faster and safer monitoring and treatment for dementia care.

## VI. Conclusion

In this paper, we propose a multi-source ensemble transfer learning approach, referred to as METL, to learn an accurate and robust classifier for the target domain. In METL, the source data sampling method ensures the transferability of samples, which are sampled from the source domain. Then, three heterogeneous classifiers are ensembled to obtain a robust classifier. Finally, multiple classifiers are combined to further improve the performance by utilizing mutual information and ensemble learning. Many experiments show that METL is accurate, effective, and robust. At the same time, METL surpasses the existing algorithms when the target training data is insufficient. AD dataset experiments prove that our approach can effectively improve the classification accuracy, solve two problems in medical datasets, and assist doctors in making a diagnosis. We propose an METL-based auxiliary diagnosis system for initial diagnosis of AD. This system helps doctors accurately identify patients in the MCI stage as soon as possible so that measures are taken to prevent or delay the occurrence of AD.
